# Contamination risk in urology operating room during the COVID-19 pandemic

**DOI:** 10.1590/1516-3180.2020.019512062020

**Published:** 2020-07-06

**Authors:** Youssef Kharbach, Abdelhak Khallouk

**Affiliations:** I MD. Assistant Professor, Department of Urology, Tangier University Hospital, Faculty of Medicine and Pharmacy, Abdelmalek Essaâdi University, Tangier, Morocco.; II MD. Professor, Department of Urology, Tangier University Hospital, Faculty of Medicine and Pharmacy, Abdelmalek Essaâdi University, Tangier, Morocco.

Dear Editor,

The novel coronavirus disease (COVID-19) caused by the SARS-CoV-2 virus is a highly transmittable and pathogenic newly emerging human infectious disease.^1^ Healthcare workers are at increased risk of coronavirus infection but they have to manage to provide optimum care to patients.

With the number of cases expected to increase in the coming weeks, we think that efficiently prioritizing surgical procedures is the first step towards facing up to the increased risks during the COVID-19 pandemic. Benign urological surgeries should be postponed until the pandemic is over.^2^ However, oncology and emergency activities should be maintained in the hospital, and healthcare teams need to become more proactive regarding respiratory hygiene, since ­SARS-CoV-2 spreads primarily through droplets.

The data concerning this disease that are available at this date remain limited. Notably, asymptomatic SARS-CoV-2 infected individuals play a role in disseminating the infection.^2^ Human-to-human spreading of SARS-CoV-2 occurs in situations of close contact with an infected person, through exposure to this person’s coughing, sneezing, respiratory droplets or aerosols.^1^

However, other routes of potential transmission are highly suspected. Viral ribonucleic acid (RNA) has now been detected in feces, thus suggesting the possibility of a fecal-oral route of transmission.^3^ Some studies have found patients that had persistently positive rectal swabs, even after their nasopharyngeal tests were negative.^3^ Moreover, Xia et al. speculated that SARS-CoV-2 may be detected in the tears and conjunctival secretions of COVID-19 patients presenting conjunctivitis.^4^ Also, viral RNA has been detected in blood, thus suggesting that infection may sometimes be systemic.^5^ SARS-CoV-2 has not been detected yet in urine, except in one study in which SARS-CoV-2 was found in urine specimens from of 6.9% of the patients and remained positive even after throat swabs turned negative.^3^^,^^6^

These potential transmission routes, i.e. both respiratory and extra-respiratory routes, may explain the rapid spread of COVID-19. Consequently, urologists should be careful in the operating room, especially with patients who are potentially infected with COVID-19.

All staff need to be specifically trained to don, doff and dispose of personal protective equipment (PPE), including masks (FFP2 or 3), eye protection, double non-sterile gloves, gowns, suits, caps and socks.^7^

Suspected or confirmed patients should be managed in a dedicated operating room equipped with a negative pressure environment, to reduce dissemination of the virus.^7^

There is a significant risk of SARS-CoV-2 dissemination due to pneumoperitoneum-associated aerosolization of particles in laparoscopic surgery. Thus, lowering electrocautery power settings as much as possible is recommended.^8^ Also, intraperitoneal CO_2_ should be extracted using the active smoke evacuation mode.^9^ Moreover, some authors have suggested that use of laparoscopy should be reserved for COVID-19 negative patients, except for cases in which the risks of laparotomy far outweigh the risks of laparoscopy.^10^

For endoscopic surgery in suspected or confirmed COVID-19 patients, complete protection against infection is recommended.^2^^,^^9^ The same precaution needs to be taken when the anus is accessible in the operating field.^9^ Healthcare workers in contact with such patients must also wear a double pair of gloves and long shoe covers.^7^

Bowel utilization, such as in enterocystoplasty, should also be careful and kept to minimum because of the presence of SARS-CoV-2 in feces.^2^^,^^3^

In conclusion, facing up to COVID-19 is challenging, and protecting both healthcare workers and patients is mandatory. Urologists need to adopt sufficient protective strategies and develop internal protocols for their operating room teams, to minimize infection risks.
